# Why Critical Thinking Can and Often Does Fail Us in Solving Serious Real-World Problems: A Three-Track Model of Critical Thinking

**DOI:** 10.3390/jintelligence13070073

**Published:** 2025-06-23

**Authors:** Robert J. Sternberg, Aurora Jo Hayes

**Affiliations:** Department of Psychology, Cornell University, Ithaca, NY 14853, USA; ajh385@cornell.edu

**Keywords:** critical thinking, analytical intelligence, love of ideas, hatred of ideas, intimacy, passion, commitment, triangular theory of love

## Abstract

This article deals with how love and hatred of ideas can influence, and often distort or suppress, critical thinking. Love and hate can serve adaptive intellectual functions, but in practice, they often manifest in maladaptive ways. The article reviews the role of critical thinking in adaptation, then discusses how love and hate can influence critical thinking. The article suggests that teaching critical thinking needs to take into account that real-world critical thinking often bears little resemblance to that shown in tests or in school. We need to teach critical thinking as it exists in the world, not in rarefied settings.

## 1. Introduction

If you are like the authors of this article, you know someone intelligent who seems, in their everyday thinking about one important problem or another, to have abandoned critical thinking entirely. The someone may be a loved one, a friend, an acquaintance, a colleague, or even oneself. They may have a decent IQ, even a notably elevated one, and yet, when it comes to one serious everyday problem or another, or perhaps several problems, you seemingly cannot get a coherent thought out of them.

Examples of failures of critical thinking by highly intelligent people are legion. Robert McNamara, who was often called the “architect of the Vietnam War,” was a graduate of the University of California, Berkeley and Harvard, and generally considered to be of staggering intellect. But even after the Vietnam War was failing badly, he committed the sunk-cost fallacy, a failure of critical thinking, and continued to advocate for the war (Murse 2018). More extreme was Ted Kaczynski, a Harvard-educated mathematics professor at the University of California, Berkeley, who became the Unabomber and destroyed other people’s lives as well as his own. William James Sidis is perhaps the most famous example of a failed individual with a prodigious IQ who ended up living an unhappy life, writing under pennames on topics such as Native American streetcar tokens (Mahony 2016). Bill Clinton, a Yale Law School graduate, was impeached during his term as the U.S. president in part because, when questioned about his relationship with Monica Lewinsky, a White House intern, he resorted to saying “it depends on what the meaning of the word ‘is’ is,” a statement that gained him no credibility with the members of the U.S. Senate who would be judging him (Noah 1998).

There are many reasons that IQ and related skills might be inadequate to the challenges of real-world problems. In the case of Sidis, he appeared to have personality and emotional issues that hindered his adjustment. Some theorists have suggested that the kind of intelligence measured by IQ tests and their proxies, which may be of substantial importance in school tasks, may not be the same kinds of intelligence that are most needed for successful real-world critical thinking (Wagner and Sternberg 1984). Examples of kinds of intelligence needed for real-world adaptation are contextual intelligence (Ceci and Roazzi 1994); interpersonal and intrapersonal intelligence (Gardner 2011); practical intelligence (Hedlund 2020); social intelligence (Kihlstrom and Cantor 2020); emotional intelligence (Rivers et al. 2020); rational thinking (Stanovich 2009); intuitive intelligence (Gigerenzer 2023); and adaptive intelligence (Sternberg 2021a, 2025a).

One might have hoped that, with rising IQs in the 20th century (Flynn 1984, 1987, 2011, 2020), people who do not exhibit critical thinking would have become scarcer. Even if IQs have been falling in some places (Bratsberg and Rogeberg 2018; Pietschnig and Gittler 2015), the fall would not seem to account for the situation in which many of us find ourselves—wondering whether a major proportion of one’s own country, perhaps as much as half the country—has gone off the deep edge. And all the time, one has been aware that this seemingly unthinking half of the country, and their friends or acquaintances, are thinking the same thing about oneself that one is thinking about them. Are they losing it? Are you losing it? Is everyone losing it?

This article argues that critical thinking as a competence is often at a level entirely different from, and often substantially higher than, its manifestation in performance. People have evolved to detect and protect themselves from threats, and two ways in which they do so is through love and hate. However, although love and hate can help one detect and counteract threats, they also can lead one to imagine threats and counteract them in ways that are irrational, regardless of one’s level of intelligence.

Threats can be either positive or negative in how they affect critical thinking. If they lead to greater vigilance and attention and prompt the individual to realize that clear thinking is needed to counter a particular threat, they will improve critical thinking. But if the threats lead to high levels of anxiety or confusion, or to avoidance behavior, they will impede the quality of critical thinking.

### What Is Critical Thinking?

Before discussing the argument of the article and the assumptions underlying this argument, it is important to get a sense of how various scholars have understood *critical thinking,* and what the similarities and differences are. According to Richard Paul, the essence of critical thinking is the study and evaluation of one’s thoughts (Elder and Paul 2008; Paul 2005; Paul and Elder 2008). Such analysis in turn improves one’s critical thinking.

According to Paul and Elder, critical thinking occurs in processing stages. The first stage is an analysis stage. People deconstruct their thinking—including its assumptions and its implications. The second stage is the assessment of critical thinking (Elder and Paul 2008; Foundation for Critical Thinking 2009). In this stage, critical thinkers assess their thinking based on its accuracy, internal consistency, clarity, precision, fairness, consistency, etc. Elder and Paul also have argued that critical thinking develops over time in chronological stages: (1) unreflective thinker; (2) challenged thinker; (3) beginning thinker; (4) practicing thinker; (5) advanced thinker; and (6) advanced thinker (see also Leon 2020).

Swartz et al. (2008) analyzed the metacognitive processes in critical thinking through the metaphor of climbing a ladder. Each rung of the metaphorical ladder brings one further into critical thinking: (1) an awareness of *what* kind of thinking is being done; (2) describing, in procedural terms, *how* the thinking is being done; (3) becoming more evaluative—is the critical thinking effective?; and (4) planning how to engage in this same type of thinking in future situations, utilizing the previous rungs. Such thinking becomes self-correcting, so that one is always improving in one’s thinking (see also Swartz 1987).

Perhaps the most complete taxonomy of critical thinking dispositions was provided by Ennis (1987, 1991, 2011). These dispositions of the individual include the following: (a) care that their beliefs are true and that the decisions that are based on these beliefs are reasonable; (b) seek alternative hypotheses, explanations, conclusions, etc.; (c) consider in a serious manner points of view other than one’s own; (d) do your best to be well informed before making decisions; (e) endorse a position only to the extent that it is supported by whatever credible information is available; and (f) be concerned about the welfare of others. The emphasis on dispositions is also found in the theoretical and empirical work of Nieto and Valenzuela (2012). Ennis also proposed critical-thinking abilities, including (a) focusing on the relevant question; (b) analyzing arguments; (c) asking questions; (d) judging the credibility of sources; (e) making material inferences.

Another major framework for understanding critical thinking is that of Lipman (1995, 1998, 2003), as exemplified by his program, *Philosophy for Children.* The program teaches young children to engage in deliberative inquiry and logical reasoning. The program provides a K–12 curriculum that sets up “communities of inquiry.” These communities are designed to foster and encourage critical, creative, and caring thinking. The results are better reasoning, comprehension, and evaluation. Lipman’s approach emphasizes how important children’s metacognitive processes are, a point underscored by the theoretical and empirical work of Rivas et al. (2022).

A major thinker in the field of critical thinking is Diane Halpern, whose book with Dana Dunn, *Thought and Knowledge: An Introduction to Critical Thinking* (Halpern and Dunn 2022), is a classic in the field and puts together in a masterful fashion much of the work that has been done in psychology over a period of many years.

What can critical thinking provide that IQ does not provide? IQ, and the related General Mental Ability (GMA) (Sackett et al. 2020) measure, among other things, crystallized intelligence, which, as measured, is largely based upon accumulation of declarative knowledge; and fluid intelligence, which, as measured, is largely based on reasoning about matters remote from everyday life, in terms of both content and the situations in which the content is encountered. The reasoning measured is usually inductive rather than deductive (Guyote and Sternberg 1981): There are no logically certain conclusions. Critical thinking has certain particularly important aspects for real-world problem solving.

First, critical thinking explicitly encompasses attitudes as well as abilities. Although one view of intelligence encompasses attitudes (Sternberg 2022; Sternberg et al. 2024a), most other views of intelligence do not. And a major problem in real-world problem solving is that people often simply do not want to think reflectively or at all deeply about problems. Rather, they accept what their religious, political, or ideological leadership, or their friends and colleagues, tell them (see Sternberg and Niu 2025a, 2025b). Their problem is not in their ability, but rather in their attitude toward thinking critically. They do not want to be bothered to think critically; often, they seem to just want to be entertained.

Second, the literature on critical thinking, historically, has focused on application to the everyday world (e.g., Ennis 2011; Lipman 1995, 1998, 2003; Sternberg and Niu 2025b). Although there are some accounts of intelligence that are oriented toward action in the everyday world (Sternberg 2025a), most of the literature has focused on performance on intelligence tests administered in settings that provide an extremely limited representation of real-world environmental contexts.

Third, the literature on critical thinking emphasizes solving deep, concrete, complex, often not fully soluble real-world problems (see, e.g., Lipman 1995; Sternberg and Niu 2025b). Thinking about intelligence, on the other hand, has emphasized psychometric tests with problems that are rather shallow, abstract, and soluble with a unique answer (see, e.g., any major existing intelligence test).

Finally, critical thinking is a matter of judgment and reflection (Sternberg and Niu 2025a). In this respect, it is in some ways closer to wisdom (Sternberg 2004) than intelligence is. Understanding critical thinking as related to, but distinct from intelligence, therefore, is important.

Before making the main argument of the article, it will be useful to lay out three assumptions that underlie the present analysis.

## 2. Assumptions Underlying the Present Analysis

Three assumptions underlie the analysis presented in the current article.

1. **The operations of critical thinking and analytical intelligence can be understood only in light of the tasks people need to solve and the environmental contexts in which they need to solve them** (Sternberg 2021a, 2025a). Critical thinking occurs as a person x task x environmental context interaction. How intelligently people operate depends greatly on task: If your adaptation and life depended on your ability to hunt wild animals and forage for edible plants, how well would you do? And how intelligently people operate also depends on the environmental context: Your ability to hunt a wild animal might depend on whether the animal was fearfully running away from you or menacingly running toward you. But the importance of task and situation is not limited to hunting/gathering cultures. In life-threatening situations—such as natural disasters or human-created disasters such as war—whether one can rise to using one’s intelligence and critical thinking maximally under stress becomes a matter of life or death.
2. **Real-world problems are qualitatively different from test problems.** The real tasks and problems we face in life look little like the problems we face on standardized tests. In particular, real problems:
  - are for high and sometimes life-changing (or, in extreme cases, potentially life-ending) stakes,
  - are emotionally complex and arousing, sometimes to the level that emotions cloud or utterly befuddle people’s better judgment, leading people to think in suboptimal ways,
  - are highly driven by environmental context, requiring people to balance many conflicting interests, and sometimes forcing people to decide whether they will respond in suboptimal ways because their fellow humans want suboptimal solutions,
  - do not typically have a single “correct” answer, but rather multiple answers, each of which is better in some ways and worse in other ways,
  - are lacking a third party to tell us that we even have a problem in need of solution,
  - often are unclear in their parameters, so that it is not certain what the problem is,
  - are often in need of a collective solution, usually by people with different backgrounds, interests, and stakes in the solution,
  - typically provide, at best, only vague paths to a solution, or seemingly no good paths at all, so that we have to create our own new path,
  - often unfold over long periods of time, and sometimes, change as we are in the midst of solving them so that the course we have taken stops working, even if it worked before,
  - often make it hard to figure out what information is needed for problem solution or where that needed information is to be located,
  - are often riddled with numerous and diverse bits of false or misleading information, with the information deliberately inserted to lead the problem solver down a garden path (Sternberg 2025a).
3. **The rewarded solution to a problem often is not the best answer in any objective sense, but rather, the solution that those in power want to reach, even if it is wrong, pernicious, immoral, or the product of corrupted thinking.** We live in a time when authorities are often driven by the demand for more power, more financial or other resources, more fame, or more revenge against those they view as having betrayed them. Human nature being what it is, many people succumb to authority, whatever its demands (Milgram 2009; Zimbardo 2008). In a world where there are so many strong and often contrary agendas, the idea that there are real-world problems to be solved that depend just on being given the problem explicitly, with a clear path to solution, and with a single “correct” solution that everyone accepts, seems almost quaint.

We now apply these assumptions in an analysis of adaptive intelligence and critical thinking as, in part, a response to threats.

## 3. The Costs and Benefits of Adaptive Behavior—Threats

Humans have evolved, in part, to be rapid threat detectors. If a wild beast, a human enemy, or a freak natural occurrence (earthquake, hurricane, tornado, volcanic eruption, avalanche, etc.) is about to assail one, one has little or no time to think. One must act. Or one is severely injured or dies and may fail to pass on one’s genes to the next generation. Evolutionary pressures aside, one learns in one’s life that threats can be anywhere and, sometimes, everywhere, and that if one does not react, often instinctively, one may forfeit one’s well-being or life. The time to react may not be immediate, but in general, with threats, to wait may be to risk one’s survival.

Adaptivity requires one to be self-protective or to risk one’s capacity to carry on and, ultimately, to reproduce. It also requires one to have mechanisms by which one will want to reproduce. That is, of course, where love and sex come into the picture (Buss 2016; Byrne and Whiten 1998; Geher et al. 2013; Miller 2001).

To produce a new generation, therefore, requires at least two things: adaptive intelligence to survive (Sternberg 2021b) and mating intelligence (Geher et al. 2013). It also requires other things, of course, such as advanced expertise in threat detection and related skills (Sternberg 2000), creativity (Sternberg 2025b), and wisdom (Sternberg 2004). All these skills can be taught (Grigorenko et al. 2002), but schools do not often purposely teach them. And, we argue in this essay, they can work against each other.

Adaptive intelligence leads one to recognize threats. Mating intelligence leads one to recognize good mating matches. In contemporary society, that often means choice of a long-term partner, perhaps of a marital partner. But a notable amount of mating is not intelligence-driven, but rather, driven by instinctive drives that take over, often despite one’s adaptive intelligence. Sex and love toward another individual often emerge not because of any intelligence, but rather, despite it.

Adaptive intelligence involves changing oneself to fit an environment, changing an environment to fit oneself, or selecting a new environment instead of one in which the individual does not fit, or no longer fits. It is a reflective process that often involves critical thinking. It requires one to have beliefs that are internally consistent and that are consistent with what is out there in the environment (Sternberg 2021b). It requires one carefully to seek out alternative views and arguments, to comparatively assess them, and to choose the best ones (Sternberg and Niu 2025a, 2025b). What is there to go wrong?

A major thing that can go wrong is that just as people fall in love with other people, or even with pets, they also fall in love with ideas; and just as people can hate other people, so can they hate ideas (Hayes 2025; Sternberg 2025c). Both love and hate are adaptive mechanisms to deal with threats. Through love, one has the protection of one or more others who will be there when one needs aid and support. Through hate, one can flee from or retaliate against threats as they arise, to conquer them before they conquer oneself. Suppose, for example, that one is threatened by an enemy. Love may lead those you love to try to protect you from the enemy. Hate may lead you to retaliate against the enemy so that they can never threaten you again. In times of a natural disaster, the first ones we may try to save are those we love. Those we hate, we may make no effort to save. Love and hate are thus part of a full repertoire of adaptive intelligence. Consider an elaboration on this idea.

Sternberg and Sternberg (2024) have proposed what they call a RELIC (Real Love in Context) theory of love, which is drawn upon here for understanding, in part, why it is that critical thinking and intelligence do not fare well when it comes to many real-world problems.

Part of the theory—called a triangular theory of love—has been cross-culturally validated in 25 countries and 37 languages (Kowal et al. 2023; Sorokowski et al. 2020; Sternberg 1997). The theory has also been found to apply very well to love of musical instruments (Sternberg et al. 2022), love of political figures (Goldberg and Sternberg 2024), love of food (Goldberg and Sternberg 2025); and love of academic and other disciplines (Hayes 2025). It is the most widely validated extant theory of love, at least in terms of empirical operations. Love and hate of ideas, like love and hate of people, can be protective. Good ideas help one adapt to the environment, whereas bad ideas can undermine one’s adaptation. Thus, love and hate can, under some circumstance, be adaptive as protective forces. But sometimes they lead one astray, and that is a theme of this article.

What do love and hate of an idea look like? And how do they connect to critical thinking?

## 4. Love and Hatred of Ideas Can Deflect or Even Utterly Decimate Critical Thinking

Critical thinking depends on thinking “straight” (Stanovich 2018). Love, however, often involves almost anything except thinking straight. There are exceptions. Sternberg and colleagues construct-validated (Sternberg et al. 2001) 26 different stories about love, one of which was a story that tried to analyze one’s love scientifically. But even that story is often subject to the distortion of the emotions and motivations of the person in love. The other stories, such as fairy-tale stories, business stories, and travel stories, were all based on imposed structures that define love according to how one chooses to define it, based on one’s personality, upbringing, and environment. Thus, love can be protective, but, for better or worse, we do not always choose the right form of protection.

According to the triangular theory of love, part of the RELIC theory (Sternberg and Sternberg 2024), love has three components: intimacy, passion, and commitment. Each has different characteristics. Here, it is argued that love applies not only to people, but also to ideas (Hayes 2025; Sternberg 2025c).

Intimacy is primarily emotional. It is characterized by emotional support, care, concern, communication, closeness, attachment, and trust. Intimate partners are very good friends—they are there for each other when they are needed.

How does the concept of intimacy apply to love for ideas? We can become highly attached to ideas, much as we can to people. Such ideas might be equality for our (or another’s) persecuted group, communism, capitalism, racial superiority (of one group over another or others), democracy, personal superiority in one or more respects, professional success, and financial success, among many others. We may feel comfortable with those ideas, supportive of and supported by those ideas, close to the ideas, attached to the ideas, and so forth.

Consider as an example just one of these ideas: communism. The idea of communism (as opposed to the practice) was a society that no longer distinguished between classes—no upper class, no middle class, no lower class—just a uniform “class” of workers whose resources would be owned collectively and to whom resources would be allocated based on need rather than societally valued contribution—to each according to their needs. Communism seemed to some a road to equality and an end to manifest differences in ownership of private property and injustices through social inequality. All citizens would own resources equally and experience equal ownership of the means of production, as well as equal sharing of the benefits of production (Marx and Engels [1848] 2008).

The idea of communism was (and, to some, still is) very attractive. It was especially attractive to many intellectuals, at least in the mid-20th century, especially but not exclusively in France (Aron 2001). Intellectuals and others found the idea of enforced equality attractive. They had seen how capitalism seemed to grind down many workers while enriching capitalists, and communism seemed to provide an antidote. It could be trusted, they felt, to take care of people according to their needs, not according to their ability to exploit others to meet one’s own desires and wants. It felt like a comfortable friend, because no one would be betrayed. Who would not want a partner whom one can trust to provide for one, good times or bad? As we know, the implementation was a far cry from the conceptualization (e.g., Zubok 2009).

Whether an idea provokes feelings of intimacy depends upon the person and their needs, much as is the case with human partners. Intimacy-provoking ideas have in common that they meet some kind of emotional need that is not being adequately met by one’s current set of ideas. Cults are one way of providing this kind of intimacy. Like the very best of friends, they have the apparent answers to one’s problems in life. Those answers may not be compelling to others, but then, human partners are not always compelling to others either. One finds ideas that meet one’s needs, and cult leaders specialize in convincing people that they, the cult leaders, have what only they and no one else can provide (Sternberg et al. 2024b). Even if the ideas they promote are somewhat ridiculous, with familiarity and repetition, those ideas may come to seem quite reasonable (Zajonc 2001). Autocrats know, for example, that repetition establishes credibility (Orwell 1950).

Passion is characterized by an intense need, longing, and overwhelming desire (Sternberg and Sternberg 2024). People who are passionate for others may obsess over them, may find themselves thinking about those others much of the time, and may feel that they cannot live without those others. They may even become addicted to those persons. For an idea to provoke passion, the individual affected—or afflicted—must feel a desperate need for the idea. For example, someone may feel passionately about communism, and indeed, the Russian Revolution, starting in 1917, was instigated in the name of communism. Some scientists who betrayed the United States, such as Klaus Fuchs, were passionate about communism and what it offered. In the United States of 2025, some ideologues, such as Steve Bannon, appear to be passionate about the idea of populism, or perhaps, what they believe populism to be (see Douthat 2025). They may believe, or say they believe, that government has served people with entrenched privilege in society, and that it is past time for those who have been ignored to rise up and demand their full rights.

Commitment to a partner represents the decision to stay with that partner over the long term, come what may. The individual plans to be with the other regardless of changing life circumstances, including challenges to the relationship, whether from the outside (e.g., discouragement of the relationship by others) or the inside (e.g., disagreements or illnesses). Commitment is cognitive in nature; it is a decision that, no matter what happens, one is in the relationship for good. Commitment to an idea is cognitive as well. It represents a reflective, often long-developed decision that an idea is one in which one can believe, come what may. Many revolutionaries develop a commitment to an idea and decide that if that idea is not realized, they will fight for it until it is. When governments become autocratic, one of the first things they do is take over the press and the schools (Free Press Unlimited 2024; Rippenberger et al. 2025). Thus, the 2025 lawsuits against the press in the United States and the concerted attacks on the most prestigious universities basically follow the autocratic playbook, however they may be intended. This is not a matter of left- or right-wing politics, but rather of government capture.

With ideas, as with persons, greater love is represented by greater amounts of intimacy, passion, and commitment (Sternberg and Sternberg 2024). The greater the love, the more one will fight for an idea, just as one will become more likely to fight for a person. All of this is well and good, if the idea is actually a good one. Sometimes, it is a great one.

Consider an example. The love that Marie Curie, the first woman to receive a Nobel Prize, felt toward her discoveries with portable X-ray machinery, radioactivity, radium, and polonium was palpable. In 1903, Curie became the first woman to receive a Doctor of Science degree in France, an achievement made possible by her continued passion and commitment to the field (Coppes-Zantinga and Coppes 1998; Rockwell 2003). Before attending university, Curie worked as a governess in Poland. She tutored other women in secret after advanced education was outlawed for women in Poland under Russian occupation (Langevin-Joliot 1998; Rockwell 2003). While at the university, Curie’s thesis on radiation led her and her husband Pierre to coin the term “radioactivity.” Her research ultimately contributed to her earning her two Nobel Prizes (Langevin-Joliot 1998; Rockwell 2003; The Nobel Prize 2018). But as a result of radiation exposure, Curie’s fight and love for her idea came at the cost of her life, a sacrifice of a kind that many romance novels idealize. To love an idea to its fullest potential is not always without its caveats.

Curie’s idea shifted society’s understanding of both chemistry and physics for the better. However, physicist J. Robert Oppenheimer, the “father of the atomic bomb,” also loved his idea of fission creating a weapons of mass destruction, but he and his colleagues appear to have changed the landscape of society for the worse. Despite three Nobel Prize nominations in physics and being hailed as one of the most powerful scientists in the American government during the 1940s, J. Robert Oppenheimer never won the coveted award (Bernstein 1982; Bethe 1968). Throughout this professional career, Oppenheimer was questioned about his loyalty to the United States (Bernstein 1982). Did he love his country more than he cared for the ethical considerations of the weapon he was tasked with creating?

Some may argue that, during his time as the chairman of the General Advisory Committee of the Atomic Energy Commission (AEC), his outward opposition to the hydrogen bomb may have hinted that his love of the field was greater than his love for the powers that sought to use his knowledge to cause destruction (Bethe 1968; Bernstein 1982). While witnessing the first atomic bomb explosion, he stated, “Now I am become Death, the destroyer of worlds” (NBC 1965). Can love and death exist simultaneously?

The creation of the atomic bomb was not merely a scientific breakthrough; it was also the “destroyer of worlds.” The love of scientific discovery can exist alongside the potential regret and even later hatred of the invention. In addressing and understanding how our love of an idea can be seen through different contextual lenses, we are better equipped to navigate the pluses but also the minuses of our ideas.

As most people learn through hard experience, love is not always directed toward others who are a good match or even deserving of the love. The same challenges apply to love of ideas. For example, some of the individuals who originally loved communism later turned from the extreme left to the extreme right, such as Whittaker Chambers (Tanenhaus 1997). As can be true with love of persons, love can turn to dislike and even hate. And when this conversion happens, hate, like love, can impair critical thinking.

One’s love of one’s ideas is not always a stable relationship. Like that of individuals, love for an idea can begin fruitfully but turn sour in a matter of seconds. Love of an idea is not merely constructed and impacted by one’s personal experience and relationship to it, but also by unifying and/or opposing ecosystems (Bronfenbrenner 2000; Sternberg and Sternberg 2024). These ecosystems, as defined by Bronfenbrenner, consist of five levels: (1) the microsystem (one’s immediate environment); (2) the mesosystem (the connections between different microsystems); (3) the exosystem (the environment one is not immediately apart of, but still impacts their life); (4) the macrosystem (the broader sociocultural context); and (5) the chronosystem (the change in environment over one’s lifetime). Just as love between individuals can falter and waver under external pressures, so can the love one feels toward an idea. As easily an ecosystem can nourish the idea, it can just as easily inhibit or potentially completely dismantle how one feels about one’s idea. One’s critical thinking can be facilitated or impaired by these factors in the environment. If, for example, one voted for a politician because one loved the politician and their ideas but then found out that what the politician said before being elected and what they do after election have little in common, one may feel betrayed; one can find oneself thinking only in negative and even hateful terms of the politician. If the betrayal is severe enough—for example, if one’s spouse is about to be deported—it becomes hard to think critically or at all objectively about the politician.

Hatred of ideas, and often of the people who cling to those ideas, can distort thinking at least as much as love of ideas can, and possibly more. Hatred, like love, can be understood in terms of three components, namely, negation of intimacy, passion, and commitment (Sternberg 2003). Hate-mongers try to cultivate all three components when they turn people into haters.

Negation of intimacy refers to feelings of aversion, repulsion, and extreme distaste. Someone who experiences the negation of intimacy toward another is repelled by them. They want to have as little as possible to do with them and to avoid any physical contact. They often find these others to arouse disgust and loathing and seek to distance themselves to the greatest extent possible. Negation of intimacy in terms of an idea involves finding the idea vile, disgusting, repulsive, or inhuman. Examples, for some people, might be eugenics, lab-grown meat, communism, capitalism, or miscegenation. The same ideas that one person hates and reviles, another might love and find to be precious.

Passion in hate represents much the same arousal as in love, but with a negative valence. Instead of feeling passionate about connecting with the loved one, one feels passionate about one’s aversion to them. Often, one sees them as a threat or as a source of dark energy of some kind; one passionately wants to either avoid them or fight against them. Revolutionary movements can be fomented and encouraged by hatred toward a governmental entity and the idea or ideas it represents, such as hatred of King George III, or taxation without representation, during the American Revolutionary War.

Commitment in hate represents the result of a cognitive investment to produce what seems like sound reasons for one’s extreme negative feelings toward another. With hatred of ideas, commitment represents a process whereby one comes to believe that one’s negative feelings are well thought out, justified, and worth retaining, even when those ideas are challenged or disconfirmed, including by a governmental system with the power to imprison or even execute one.

For example, cult leaders and their affiliated members operate under the assumption that all ideas that contradict the teachings they are trying to disseminate to their followers are both incorrect and harmful. Through the systematic erosion of critical thinking, cult members come to love their ideas so much that their hate for outsiders may become an emotion equally contributing to their decision-making (Langone 1993). The 1997 mass suicides of 39 members of the Heaven’s Gate cult illustrate how far our love of an idea and our eventual hate of others’ ideas can lead the critical thinking of the citizens of a society to become warped beyond repair (Balch and Taylor 2002). As a reminder, Heaven’s gate was a UFO cult that began during the 1970s and resurfaced during the 1990s; followers believed that the comet Hale-Bopp was used as a disguise for an alien spacecraft. In March 1997, when the comet was at its closest to Earth, members of the group drank a lethal elixir of drugs. Their hope was to leave their bodies, enter the alien spacecraft, and exit into Heaven’s Gate (the celestial plane). Before taking the action that he hoped would lead himself and his followers into the celestial plane, the leader of Heaven’s Gate, Do, said, “We are returning to life and we do in all honesty *hate* this world” (Balch and Taylor 2002).

In the case of the members of Heaven’s Gate, their love of their ideas was not just a means of existence; they were also the fruits that would lead to their ultimate end. The power of love and hate cannot be underestimated in their contribution to how we digest information and later act on what we have received. Do and his followers believed that they had exhausted all human options to escape the inevitable end of the world (Balch and Taylor 2002). Their disdain for outsiders was, in their belief, a well-thought-out and justified reaction to non-members’ lack of belief in their cause. When love and hate fuel one another to neglect reality, our decisions no longer feel as though they are our own, but rather, exist to promote the idea. Unfortunately, people in cults generally do not see themselves as being in cults, so they do not perceive the impairment in their critical thinking. The cult members may come to think that they (those in the cult) are the only ones who think critically.

Love and hate of ideas can be every bit as powerful as love and hate of persons. People die in the service of ideas, just as they die in the service of other people. Nathan Hale’s reputed famous last words, before he was hanged by the British—”I only regret, *that I* have but one life to lose for my country,” represented love of country and the ideals upon which it was founded. In his case, one might believe that his love of country was well thought through and justified.

In recent times, North Korean soldiers have been fighting and dying in Ukraine, having been recruited by their government to serve the Russian one in a genocide against Ukraine. During World War II, German and other soldiers died in the service of Germany and the Nazi movement it spawned. It is perhaps harder to justify service to one of the most reviled governments in the world (North Korea—Pianin 2017) or one of the most reviled ideological movements in history (Nazism). People can be indoctrinated, it appears, to serve and even love almost any cause, no matter how horrific, and then to act in a self-justificatory way (Bandura 2015; Milgram 2009; Zimbardo 2008). Indoctrination can serve as a defense against criminal charges (Robinson and Holcomb 2020), but the strong whiff of immorality for the actions undertaken never goes away. People fall in love with ideas or countries that embody immoral ideas, no matter how attractive or unattractive they may be to others and to posterity.

A problem is that people develop myside biases—biases in favor of whatever their current ideas may be—and intelligence and rational thinking not only appear to be largely powerless to combat them but even can serve those biases by giving people elaborate rationalizations for the beliefs they favor (Baron 2023; Stanovich 2021), or, as discussed here, love. So can develop, as Stanovich has noted, the biases that divide us. Many highly intelligent people, including *Communist Manifesto* authors Vladimir Lenin and Friedrich Engels, have fomented and served causes that have proven to be toxic.

Love and hatred of ideas can be found even in the most mundane circumstances. Although we used high-stakes and historically prominent examples to illustrate these phenomena, it is equally important to recognize that passion for an idea can develop from the simplest and most common experiences. For many academics, medical professionals, writers, historians, etc., their love for their profession began with what others would consider an ordinary event. Whether that be a subject in school that fueled their imagination, a book that sparked their curiosity, or an invention they imagined could change the world, that love began with an interest. This initial interest could come to fuel a love toward the idea, or, in some cases, it might lead to hate. Even the later disdain Dr. Oppenheimer felt toward his (partial) creation, the atomic bomb, began with a passion for physics. One’s affective response to one’s idea(s) can begin anytime, anywhere, and with any subject. As one comes to love an idea more and more, or to hate it more and more, one’s critical thinking may suffer, as would be the case for love of, or hatred toward, a person.

## 5. A Three-Track Model of Critical Thinking

**Figure 1:**
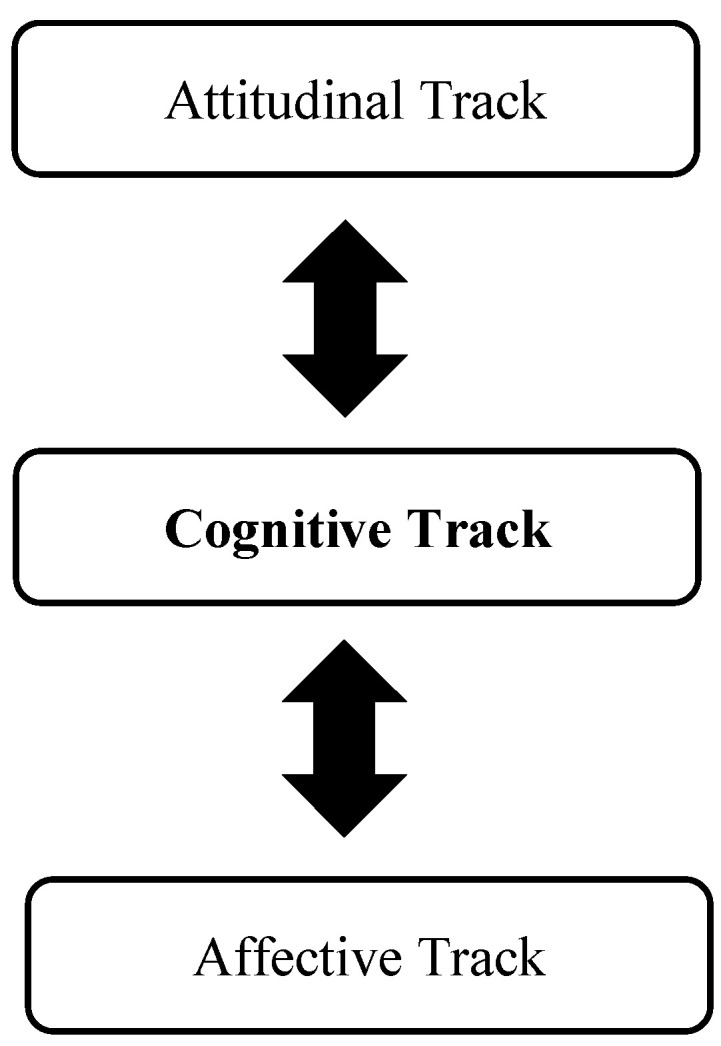
Three tracks of critical thinking.

Based on the considerations presented in this article, we propose a three-track model of critical thinking. The model is summarized in Figure 1. The three tracks in the model are a cognitive track (which is the primary track), attitudinal track, and affective. This model is not intended as a wholly new perspective on critical thinking, but rather as a model that integrates many existing strands of thought about critical thinking, as discussed earlier.

The model suggests that critical thinking is primarily cognitive (bolded print in Figure 1), but it is also continually interacting with the attitudinal track and the affective track (which also includes personality states and traits). The attitudinal and affective tracks can either facilitate or impede the quality of critical thinking. For example, an attitude of cognitive inertia or of myside bias will impede critical thinking, the former by preventing one from instigating critical thinking, the latter by allowing critical thinking but then by biasing it once it occurs. An affect of love for one’s work may increase one’s engagement in critical thinking, whereas hate for the work one does may decrease one’s engagement. Love may increase engagement but also lead to biased thinking.

Table 1 presents the model in somewhat more detail. There is no attempt to be exhaustive with respect to all the attitudinal, cognitive, and affective states and processes that can influence critical thinking. Rather, the table presents a sample of the kinds of states and processes that can influence the outcomes of critical thinking. As the table shows, attitudes and affects can influence critical thinking in either a positive or a negative way.

## 6. Implications for Education

The proposed model of critical thinking has some implications for education that we hope schools might take into account in that so many students seem to lack adequate critical thinking in these times.

1. **Critical thinking does not come naturally.** Critical thinking involves complex metacognitive and cognitive processes integrated with attitudinal and affective variables that can facilitate or impede it. Teachers cannot assume that students will just learn how to do it by being in school or by being on their own. Many students graduate from school and are nevertheless deficient critical thinkers.
2. **Critical thinking taught in the abstract as a set of metacognitive and cognitive processes is inadequate to meeting the demands of the everyday world.** As soon as people have a vested interest in an outcome or a feeling of personal or ideological alignment with a certain viewpoint, their critical thinking will begin to be affected by the alignment. Part of instruction needs to be teaching students to be aware of their own biases and counteract them.
3. **Much of critical thinking is determined, just as the critical thinking gets seriously started, by what problems one recognizes and how one defines those problems.** So much of problem solving is a matter of how one defines problems. That is why, say, Vladimir Putin refers to the invasion of Ukraine as a “special military operation” instead of, say, a genocide aimed at wiping out a separate Ukrainian identity. Or why people who view abortion as a matter of “right to life” usually come to conclusions different from those who define abortion as a matter of “women’s choice with their own bodies.”
4. **People often use their analytical (IQ-based) intelligence not to improve their critical thinking but rather to garner support for their own prior position.** High IQ can help critical thinking by improving metacognitive (metacomponential) functioning, but it is at least as likely merely to serve as a means for people to figure out ever more clever reasons to support their own position—much as in debate contests.
5. **Standardized testing could, but generally does not, help support critical thinking.** Students growing up in a testing culture learn, very often, not how to think critically but rather how to provide authorities with the answers that the test-taker thinks the authorities want to hear. Thus, standardized testing may discourage critical thinking in favor of learning how to produce ingratiating responses.
6. **Critical thinking has both domain-general and domain-specific aspects.** Because abilities, attitudes, and affects all influence critical thinking, the quality of critical thinking may vary greatly across domains as a function of one’s interests, ideologies, abilities, and efforts. At the same time, the metacomponential executive processes are largely the same across domains, so there is some domain-generality as well.
7. **One cannot improve critical thinking if one requires it of others but does not show it oneself.** Students and everyone else acquire much of their tacit knowledge base by observational learning (Bandura 1986). Ultimately, as Bandura showed, people will model the behavior they observe far more than they will base their behavior on what they are told.
8. **Critical thinking is desperately needed in today’s world, but the current emphasis on knowledge acquisition often generates students who lack the critical thinking skills they need to succeed in the world and also to make the world a better place.** Teaching for facts may lead to success on achievement tests that superficially measure school achievement, but it will not lead to success when students need to confront real problems in real-world contexts.
9. **Love can either fuel or detract from critical thinking.** As educators, we need to ensure that students are aware of how an emotion such as love can yield critical thinking. Love, especially passionate love of an idea, can lead to great advances in creativity and knowledge. But it also can lead to the same kind of distorted or even obsessive thinking that people in love sometimes feel toward people with whom they fall in love, especially in the early stages of a romantic relationship.
10. **Recognizing the connection between the affective and attitudinal tracks of critical thinking is imperative to helping students understand that their ideas may not always remain unchanged**. Critical thinking is a process that takes time, practice, patience, and care. One’s ideas may take on new meanings and evolve year to year or perhaps even day to day. Teaching students to understand that their critical thinking is impacted by the positive but also the negative effects of the attitudinal and affective tracks may fundamentally reshape their understanding of the idea at hand. This reshaping is not a phenomenon to fear but rather a testament to the continued pursuit of engaging in critical thinking.

## 7. Conclusions

When we talk about measuring and teaching for “critical thinking,” we are often talking about critical thinking in the abstract—as reflected in performance on tests and in school. The problem is that such critical thinking can be and often is bypassed by the vagaries of real life—our ideology, our religious or political beliefs, and our vested interests in particular ideas. We love others through stories (Sternberg and Sternberg 2024). We do not actually know the other, only the story through which we filter our knowledge of the other. Sometimes, the stories we create change very rapidly, as when we discover a betrayal or a piece of what we consider to be compromising history that the other failed to disclose. In an instant, love can turn to hate, or at least, intense dislike. The turning may feel as though it is rational, but as likely as not it is a result of one story replacing another—a story we do not like with one we liked.

Critical thinking, in the present view, is not merely a matter of cognitive abilities (see Figure 1 and Table 1). It also involves, inevitably, dispositions and attitudes. Moreover, its execution is influenced by affective states as well as personality traits. If one does not want to think critically—if one experiences too much cognitive inertia—one will not think critically, no matter the level of one’s abilities. Furthermore, critical thinking is very much influenced by affective variables, such as love and hate. To our knowledge, this is the first account of critical thinking that draws on theories of love (Sternberg and Sternberg 2024) and hate (Sternberg 2003) to show that critical thinking in practice cannot be separated from the affective components that influence it. When you love or hate ideas—or people or things about which you think—your critical thinking most likely will be affected, often for the worse.

Every theory of critical thinking is different. If we were to characterize what distinguishes ours from many other models, we would point to three highlights: (a) the specification in the model (as depicted in Figure 1 and Table 1) of the interaction between cognition, on the one hand, and attitudes and affect, on the other; (b) the particular specification of the metacomponents of critical thinking, which derive from the theory of adaptive intelligence; and (c) the use of a particular theory of love—the triangular theory (and also of hate)—to understand how affect can influence critical thinking.

Schools do not seem to teach development of critical thinking, or at least, not as much as some might wish. PISA scores in reading, mathematics, and science have shown a generally declining pattern in the Western world (Colombatto 2024), suggesting that knowledge and the ability to reason with it are moving in the wrong direction in the student body of the Western world. Teaching critical thinking would be a valuable first step in improving the critical thinking and analytical intelligence of students. Students can be taught these skills (Grigorenko et al. 2002), but teaching in ways that ignore the importance of real-world forces that lead students to love ideas, hate ideas, or anything in-between, is not likely to result in meaningful improvement in critical thinking.

Authoritarian governments in the world are progressing full speed ahead, both in getting elected and in staying in power (Repucci and Slipowitz 2022). In 2025, the United States, a country with a long history of democracy, is heading toward authoritarianism at a dizzying pace, literally day by day, according to hundreds of government scholars (Langfitt 2025). Such a government works in favor of collaborators who benefit from it but limits the rights of many others. In a country where many individuals seem to have reduced or suspended critical thinking (Well 2023), the results may not be particularly concerning, but the long-term result is the decline of democracy and its gradual replacement with autocracy (Albright 2018; Mounk 2018, 2023; Ziblatt and Levitsky 2018). This is not a matter of preference for one political party or another, or for right- or left-wing governments. Autocracy can originate on the right or the left, and with any political party. It is a matter of change of a form of government, democracy, that has been in existence, however imperfectly, since 1787. Autocracy, and toxic leaders in general, can be very attractive, especially to people who feel victimized (Lipman-Blumen 2006; Sternberg et al. 2024b) or who see ways to use the autocracy for personal enrichment or other gain. Countries can decide to change their form of government, but often, when they elect toxic leaders, the people do not realize that they will be electing not only a new leader but inadvertently ushering in a new form of government. And this is why critical thinking is so important in real life, not just in the taking of standardized or other tests. High IQ will provide no protection against lapses in critical thinking due to myside bias, and may actually encourage such lapses when people of high IQ think they are immune to failures in critical thinking (Stanovich 2021).

Although critical thinking and intelligence (as tested) are related, they are not the same, as noted throughout this article. A question arises as to whether higher measured intelligence might lead to *more* use of critical thinking, because intelligent people should recognize the importance of critical thinking, or whether those with higher measured intelligence might use *less* critical thinking because smart people trust their intuitions and do not believe that they need to think things through carefully. We believe that whether higher intelligence leads to more or less critical thinking depends not on intelligence or even on critical thinking, but rather, on a central aspect of wisdom, namely, epistemic humility (Grossmann et al. 2020; Sternberg 2024). Epistemic humility, as we conceive of it, is understanding what one knows, what one does not know, what one can know, and what one cannot know (Sternberg 2024). In this case, an intelligent person might believe they know what they do not know—that they are generalized experts—and thus not use critical thinking when it is needed; or they might recognize how much they do not know but that they could know and then use critical thinking. As a result, an intelligent person lacking epistemic humility can be dangerous, because they act in ways that fail to reflect their understanding of their own ignorance.

Students need to learn not only how to think critically, but how their attitudes and personal likings, loving, and hatreds can inadvertently alter their critical thinking. They need to understand that no matter what ability they have to think critically, in real life, there is a gap, often a huge gap, between competence and performance (Sternberg 2025a). No matter how intelligent we are, we are often unaware of the competence/performance gap. We feel that we are operating at a level of full competence, even when we are not because of the influence of attitudes and affects. This is scarcely a new observation. Plato and Aristotle both wrote about how the passions can influence our thinking. Perhaps, after all these centuries, it is time to take their message seriously.

## Figures and Tables

**Table 1:** A three–track model of critical thinking.

I. Cognitive Track (Metacomponents)			
Recognition of Problem Definition/Analysis of Problem Acceptance of Problem Mental Representation of Problem Allocation of Resources for Problem Solution Formulation of Strategy for Problem Solution Monitoring of Solution Strategy Evaluation of Solution			
**II. Attitudinal/Dispositional Track**			
		*Positive Effects*	*Negative Effects*
Information Seeking		Adequate Information	Inadequate Information
Desire to Think Analytically/Critically		Deep Analysis	Superficial Analysis
Willingness to Adopt Multiple/Alternative Perspective		Multi-Perspective Analysis	Uni-Perspective Analysis
Willingness to Question One’s Own or Others’ Solutions		Questioning of Solution	Uncritical Acceptance of Solution
Caring If Solution Is Optimal		Optimizing	Satisficing
Willingness to Think “Outside the Box”		Creative Solution	Pedestrian Solution
Asking: Optimality for Whom?		Common-Good Solution	Egocentric-Good Solution
**III. Affective Track**			
		*Positive Effects*	*Negative Effects*
Love			
	Intimacy	Familiarity with Problem and Requirements	Entrenchment in Solving Problem
	Passion	Burning Desire for a Solution	Positively Motivated Distortion
	Commitment	Will See Problem through to the End	Cognitive Commitment to Positive Distortion
Hate			
	Negation of Intimacy		Desire to Distance/Separate from Agents
	Passion		Negatively Distorted Motivations
	Commitment		Cognitive Commitment to Negative Distortion

## Data Availability

No new data were created or analyzed in this study.
